# Alzheimer Disease Blood Biomarker Concentrations Across Race and Ethnicity Groups in Middle-Aged Adults

**DOI:** 10.1001/jamanetworkopen.2025.45046

**Published:** 2025-11-21

**Authors:** Adam M. Brickman, Chandra Muller, John Robert Warren, Eric Grodsky, Soobin Kim, Michael J. Culbertson, Bharat Thyagarajan, Jennifer J. Manly

**Affiliations:** 1 Taub Institute for Research on Alzheimer’s Disease & the Aging Brain, G.H. Sergievsky Center, Vagelos College of Physicians and Surgeons, Columbia University, New York, New York; 2 Department of Neurology, Vagelos College of Physicians and Surgeons, Columbia University, New York, New York; 3 Department of Sociology, University of Texas at Austin; 4 Institute for Social Research and Data Innovation, University of Minnesota, Minneapolis; 5 Department of Sociology, University of Wisconsin-Madison; 6 Center for Demography of Health and Aging, University of Wisconsin-Madison; 7 Department of Laboratory Medicine and Pathology, University of Minnesota, Minneapolis

## Abstract

**Importance:**

The incidence and prevalence of clinical Alzheimer disease (AD) are higher among Black and Latinx older adults than among White older adults. Past studies that compared plasma AD biomarker concentrations among groups minoritized by their race and ethnicity yielded inconsistent findings; however, these efforts did not include population representative samples or statistical procedures to ensure population representation.

**Objective:**

To examine race and ethnicity differences in plasma AD biomarker concentrations and in the association between biomarkers and medical conditions in a US population-representative cohort of middle-aged adults (approximately 58 years of age).

**Design, Setting, and Participants:**

Data for this cohort study came from the High School and Beyond sample, a nationally representative cohort of high school sophomores and seniors who were recruited in 1980. In 2021, a subset of participants provided blood samples that were assayed for amyloid-β (Aβ42/Aβ40 ratio), phosphorylated tau-181 (pTau-181), neurofilament light chain (NfL), and glial fibrillary acidic protein (GFAP). The analyses for the present study were conducted between July 2, 2024, and August 26, 2025, using data collected during the 2021 follow-up study.

**Exposures:**

Race and ethnicity groups and common medical conditions.

**Main Outcomes and Measures:**

General linear models with Wald tests were used to compare biomarker concentrations between race and ethnicity groups and to test interactions with common medical conditions using unadjusted biomarker values and models adjusted to ensure population representation with inverse probability weighting and multiple imputation.

**Results:**

The sample included 4340 adults (mean [SD; range] age, 58.1 [1.1; 56-63] years; 2400 [55.3%] women); 630 (14.4%) were Black, 900 (20.7%) were Latinx, and 2610 (60.1%) were White. Black participants had a lower Aβ42/Aβ40 ratio (*d* = –0.002; 95% CI, –0.004 to –0.000; *P* = .04) and lower NfL concentrations (*d* = –1.16; 95% CI, –2.15 to –0.16; *P* = .02) than White participants, but these differences were attenuated when models were adjusted for population representation (*d* = 0.000; 95% CI, –0.002 to 0.002; *P* = .85 for Aβ ratio; *d* = −0.88; 95% CI, –1.78 to 0.02; *P* = .05 for NfL). Latinx participants had lower GFAP concentrations than White participants (*d* = −3.87; 95% CI, –7.30 to –0.45; *P* = .03), but these differences were also attenuated when models were adjusted for population representation (*d* = 3.36; 95% CI, –3.13 to 9.86; *P* = .31). In general, estimated biomarker means were similar between race and ethnicity groups. History of type 2 diabetes was associated with increased NfL concentration (*d* = 0.19; 95% CI, 0.07 to 0.30; *P* = .04), and high body mass index was associated with lower Aβ42/Aβ40 ratio (*d* = −0.13; 95% CI, −0.21 to −0.06; *P* = .02); whereas high cholesterol was associated with lower pTau-181 concentration (*d* = −0.18; 95% CI, −0.25 to −0.10; *P* = .01) and high BMI was associated with lower GFAP concentration (*d* = −0.30; 95% CI −0.44 to −0.16; *P* = .01). No differences in associations between morbidities and AD biomarker concentrations were detected across race and ethnicity groups.

**Conclusions and Relevance:**

In this cohort of middle-aged adults, the use of appropriate statistical estimation to ensure population representation indicated that blood-based AD biomarker concentrations were not distinguishable among race and ethnicity groups. Common medical conditions were associated with plasma biomarker concentrations similarly across race and ethnicity groups. These results highlight the importance of considering population representation and comorbid conditions in AD research to ensure accurate characterization of disease pathophysiology and improve precision of diagnostic and treatment strategies for populations that experience AD disparities.

## Key Points

**Question:**

Do the concentrations of plasma biomarkers associated with Alzheimer disease differ among race and ethnicity groups in middle-aged adults?

**Findings:**

In this cohort study of 4340 adults approximately 58 years of age in the High School and Beyond study, compared with White participants, Black participants had lower amyloid-β (Aβ)42/Aβ40 ratios and lower neurofilament light chain concentrations, and Latinx participants had lower glial fibrillary acidic protein concentrations, but these differences were attenuated when statistical techniques were applied to ensure population representation. Common medical conditions, including type 2 diabetes, high cholesterol, and high body mass index, were associated with biomarker concentrations, but these associations did not differ across race and ethnicity groups.

**Meaning:**

This study found that Alzheimer disease–associated biomarker concentrations were similar among race and ethnicity groups for middle-aged adults when sampling and statistical modeling techniques were used to ensure population representation.

## Teaser

This cohort study investigates race and ethnicity differences in plasma concentrations for biomarkers of Alzheimer disease and assesses the association between these biomarkers and medical conditions among middle-aged adults.

## Introduction

Over the past several decades, the in vivo diagnosis of Alzheimer disease (AD) has evolved from one based on consideration of symptomatology exclusively^1^ to one based on evidence of the primary pathophysiological processes of the disease irrespective of symptoms.^2^ This change in diagnostic conceptualization parallels development of the biomarkers used to operationalize AD pathophysiology. Initially, pathological features of AD could only be appreciated with direct examination of brain tissue, but molecular positron emission tomography and analysis of cerebrospinal fluid allowed for the measurement of AD-related protein aggregates and concentrations.^3^ More recently, high-sensitivity immunoassays were developed to detect very low concentrations of AD-related proteins in the blood compartment, including amyloid-β (Aβ), phosphorylated tau, neurofilament light chain (NfL), and glial fibrillary acidic protein (GFAP), which capture the amyloid, tau, and neurodegeneration cascade associated with AD pathogenesis and course.^4^

Blood-based biomarkers for AD hold great potential in a variety of contexts. Clinically, assessment of AD pathophysiology through a simple blood test opens the possibility of diagnosis, disease monitoring, and evaluation of treatment eligibility in primary care settings without the need of specialty clinics. From a research perspective, lumbar puncture and molecular positron emission tomography imaging are not always feasible due to cost, access to specialized technology, and participant acceptance. Incorporation of blood-based biomarkers in research allows for the evaluation of factors that influence the biology and clinical expression of AD across diverse groups with high fidelity and relatively low cost. Research conducted with AD-related blood-based biomarkers has focused primarily on establishing measurement validity and identification of diagnostic cut points by contrasting those with or without prevalent disease.^5^

The incidence and prevalence of clinical AD and related dementias (ADRD) differ widely across race and ethnicity groups in the US, with well-documented increased risk among Black and Latinx older adults.^6,7^ These differences are shaped by social and structural inequalities rooted in systemic racism.^8^ Despite the greater AD-related burden experienced by these racial and ethnic minoritized groups, they are notably underrepresented in most biomarker studies. Direct comparison of plasma concentrations among race and ethnicity groups have yielded inconsistent findings, with some reporting differences in plasma biomarker concentrations among Latinx, non-Latinx Black, and White adults^9,10,11,12,13,14^ and others reporting similar biomarker levels, particularly after adjustment for confounding variables.^15,16^ Reported differences in biomarker concentrations between race and ethnicity groups in these studies indicate less severe degrees of pathophysiology among minoritized groups in middle age and late life than among White people, although findings are notably inconsistent across studies with respect to both directionality and specific biomarker,^10^ raising the possibility that other factors besides AD pathophysiology per se contribute to disparities in ADRD. In our review of the literature, racial and ethnic group differences are more likely reported in clinic-based or convenience samples than in community cohorts or cohorts recruited to be population representative, suggesting that sampling bias may contribute to positive results. Establishing whether biomarker concentrations differ among race and ethnicity groups is paramount, as previous authors, guided by purported differences among groups in biomarker concentrations, recommended adjusting diagnostic biomarker cut points by race or ethnicity^17^ or that the biology of cognitive impairment fundamentally differs by race,^18^ conclusions with profound implications for diagnosis and treatment of AD.

In the current study, we used data from the High School and Beyond (HS&B:80) cohort to examine race and ethnicity differences in blood-based AD biomarker concentrations among adults approximately 60 years of age. HS&B:80 data were collected in 2021 from people who were first observed when enrolled in high school in the United States in 1980 and who were reinterviewed through early adulthood and in midlife. The initial sample was drawn such that every person enrolled as a sophomore or senior in a high school in the United States in 1980 had a known, nonzero probability of inclusion. These probabilities were converted to statistical weights and applied to analyses to produce estimates of population-representative statistics. We considered 2 major questions in this work. First, do AD plasma biomarker concentrations vary by race and ethnicity in the population of US adults who were high school sophomores or seniors in 1980? Second, what are the medical and demographic correlates of biomarker concentrations and do they vary by race and ethnicity group? Addressing these questions in this age stratum is critical because the emergence of disparities and potential impact of modifiable factors are greatest in midlife.^19^ While our overall hypothesis was that, with appropriate weighting for population representativeness, there would be no differences in AD biomarker concentrations across racial and ethnic groups, we anticipated that medical and genetic (ie, *APOE* genotype) correlates would differ among groups, reflecting the various potential pathways that shape AD risk.

## Methods

This cohort study was reviewed and approved by the NORC (formerly the National Opinion Research Center) at the University of Chicago and University of Minnesota institutional review boards. Written informed consent was obtained when participants were interviewed for the survey and agreed to have a home health visit at which blood would be drawn. This study follows the Strengthening the Reporting of Observational Studies in Epidemiology (STROBE) reporting guideline for cohort studies.

### Sample

The HS&B:80 study launched initially in 1980 and comprised a nationally representative probability sample of 30 030 high school sophomores and 28 240 high school seniors (mainly born between 1961 and 1965) from a probability sample of 1020 US public and private high schools.^20^ A random subset of 14 830 sophomores and 12 000 seniors from the initial sample of 58 270 students was interviewed and evaluated at multiple time points through 2021. Results from this study were derived from 4340 panelists evaluated in 2021 who provided blood samples. All sample sizes are rounded to the nearest 10 per the restricted use data licensure requirements of the US Department of Education, National Center for Education Statistics, and all reported percentages are based on these rounded values.

A procedural overview of the HS&B:80 study was described in detail previously.^21,22^ Briefly, the 1980 survey collected information about educational experiences, school curriculum, school composition, student outcomes, and socioeconomic background gathered from students, parents, teachers, and school administrators. Students additionally completed multiple-choice achievement tests. Follow-up surveys were conducted for senior and sophomore panel members in 1982, 1984, and 1986; for sophomores only in 1992 and 2014; and for seniors only in 2015, with response rates from roughly 90% in the 1980s to approximately 65% in 2014 and 2015.

**Figure 1.**
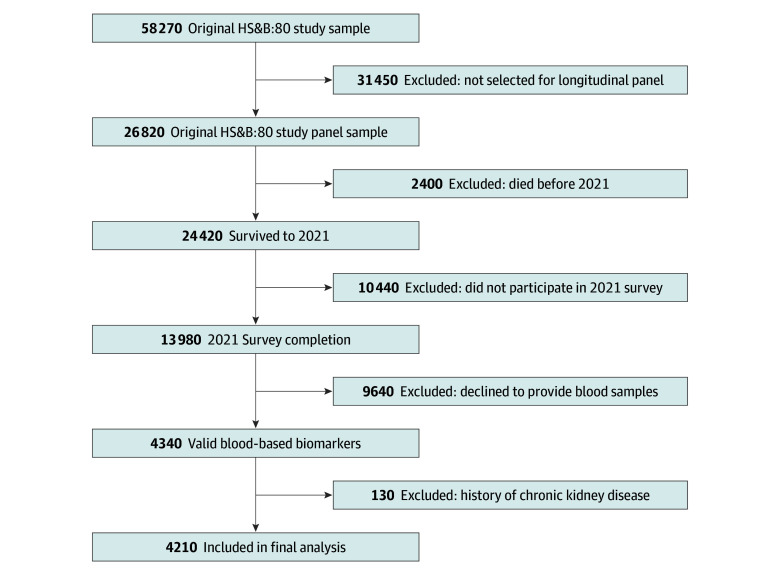
Flow Diagram Depicting Selection of Participants in the Current Analyses HS&B:80 indicates High School and Beyond 1980. SOURCE: Author calculations are from the US Department of Education, National Center for Education Statistics, The High School and Beyond 1980: 2021 Follow-Up. Sample sizes are rounded and based on unweighted numbers.

In 2021, surviving cohort members were recontacted for a follow-up interview either by telephone or the internet. This was the first wave of data collection in which health and cognition were the primary focus; previous waves of data collection focused on educational attainment, family, and labor market transitions and experiences. All individuals who participated in a 2021 follow-up interview were invited to provide blood samples during a separate in-home visit by a phlebotomist. Figure 1 summarizes the restrictions of the original HS&B:80 study sample to the 2021 sample of blood-based biomarkers by mode.

### Demographic and Medical Variables

Demographic variables were ascertained by self-report during the 1980 and 1982 surveys and 2021 follow-up visit. Race and ethnicity were considered as 4 mutually exclusive categories: Hispanic or Latinx (Latinx), non-Hispanic/Latinx Black or African American (Black), non-Hispanic/Latinx White (White), or other, which included any other race or ethnicity. Sex was ascertained by self-report using a binary response in 1980.

We considered a number of medical comorbidities based on previous reports,^13^ including chronic kidney disease (CKD), hypertension, type 2 diabetes, heart disease, and dyslipidemia or high cholesterol. For all conditions, apart from high cholesterol, participants were asked whether a health professional ever told them that they had any one of these conditions; we assigned a score of 1 to affirmative responses and 0 to negative responses. Individuals were characterized as having high cholesterol (≥240 mg/dL) or normal cholesterol (<240 mg/dL) based on total cholesterol measurements from a blood lipid panel (to convert total cholesterol levels to millimoles per liter, multiply by 0.0259). Additionally, we calculated body mass index (BMI) from self-reported height and weight using the formula [(weight in pounds)/(height in inches)^2^] × 703. Participants were categorized as having low BMI (<18.5), normal BMI (between 18.5 and 25.0), elevated BMI (between 25.0 and 30.0), or high BMI (>30.0). We considered measurement of creatine as a continuous assessment of kidney function. *APOE* genotyping was performed by using a TaqMan SNP Assay (Thermo Fisher); we categorized individuals dichotomously as having 1 or 2 copies of the *ε4* allele or as having no copies of the *ε4* allele.

### Biomarkers

Trained phlebotomists drew 4 serum separator tubes and 3 10-mL EDTA tubes at participants’ homes. The serum tubes were centrifuged prior to shipping, whereas the EDTA tubes were shipped via overnight service to the Advanced Research and Diagnostics Laboratory at the University of Minnesota for aliquoting and analysis. A vast majority of the samples were processed, aliquoted, and stored within 48 hours of collection. Immunoassays were conducted on frozen EDTA plasma and serum plasma samples.^23^ Plasma samples were analyzed using the Neurology 4-Plex E kit (Quanterix Inc) for Aβ40, Aβ42, NfL, and GFAP. The ratio of Aβ42 to Aβ40 was calculated as a measure of amyloid pathophysiology. Serum samples were used to analyze phosphorylated tau at threonine 181 (pTau-181) using the Human pTau-181 Advantage V2 assay kit (Quanterix Inc). We selected these biomarkers because they were considered state-of-the-art for AD-related pathophysiology at the time of the blood analysis. Both plasma and serum samples were analyzed using a Simoa HD-X Analyzer (Quanterix Inc). Based on the results of a pooled plasma sample analyzed across 55 analytical runs, the coefficient of variation for Aβ40 was 4.8%; for Aβ42, 4.6%; for NfL, 9.6%; and for GFAP, 6.5%. The coefficient of variation for pTau-181 using a pooled serum control across all 55 runs was 6.1%.

### Statistical Analysis

We generated descriptive statistics to examine the frequencies and distributions of demographic, medical, and biomarker variables in the total sample and by race and ethnicity. All analyses were conducted between July 2, 2024, and August 26, 2025, with Stata, version 19.5 (StataCorp LLC). A 2-sided value of *P* < .05 was considered statistically significant.

#### Sample Weighting

The final biomarker sample differed from the population of high school students in 1980 in part due to intentional oversampling in the base year of schools with high populations of Cuban students and Catholic schools with higher proportions of Black students. Further sampling of students for inclusion in the panel included oversampling those who did not complete high school from the sophomore cohort and Black students with relatively high propensities to attend college based on their academic performance, sample attrition, and declination to participate in the blood draw. We used a combination of 2 techniques to adjust the biomarker sample to represent the intended population. To account for differences between the HS&B:80 study panel sample and the population due to sample design (including the stratification of schools in the base year and probabilistic selection of students for the panel), we weighted all cases in the full panel sample with the inverse probability of selection.^24,25^ These probabilities were known due to the study sample design. To account for differences between the 2021 blood draw subsample (N = 4340) and the full surviving study sample (N = 24 420) due to nonrandom sample attrition and declination to provide a blood sample, we used multiple imputation^26,27,28^ with chained equations^29^ to impute missing biomarker and covariate values. This imputation consisted of taking the expectation of estimated quantities over the distribution of missing values, given the known information about each sample member (eTable 1 in Supplement 1), and augmenting each value with a random draw from the posterior sampling distribution. Multiple imputation can provide greater estimation precision than inverse probability weighting when auxiliary information is available,^26^ such as in HS&B:80. We used 100 imputed datasets to reduce Monte Carlo error to a negligible level.^30^ Imputation diagnostic plots are presented in eFigure 1 and eFigure 2 in Supplement 1.

The resulting sample was representative of the population due to 2 design elements. First, as already noted, every member of the population had a known, nonzero probability of inclusion in the sample. Second, while sample attrition reduced representativeness, we had extraordinarily rich data from multiple points in the life course with which to model plausible values for attributes of individuals who did not respond to a particular wave of data collection. Together, these design elements helped minimize bias in the estimated statistics we present with respect to population parameters.

#### Race and Ethnicity Group Comparisons

CKD is a known confound of ADRD biomarker measurements.^13^ Prior to hypothesis testing, we confirmed an association between CKD, indexed with reported history and creatine levels, and biomarker concentrations. After confirming the association (eFigure 3 in Supplement 1), we removed 130 individuals with a self-reported history of CKD from subsequent analyses. In supplemental analyses, we also reran the primary analyses with individuals reporting a history of CKD included. We estimated a series of general linear models first to examine differences in biomarker concentrations among race and ethnicity groups with or without adjustment for population representation. Group differences were tested pairwise using Wald tests.

#### Associations of Demographic Variables and Medical Comorbidities With Biomarker Concentrations

For each demographic variable (apart from race and ethnicity) and medical morbidity, we estimated general linear models that included an interaction between the exposure of interest and indicators of race and ethnicity group. We assessed whether the association between a given biomarker and exposure varied across race and ethnicity groups using a multivariate Wald test for the interaction terms in the linear model. We used the Benjamini-Hochberg procedure^31^ to limit the false discovery rate to 5%.

## Results

The sample included 4340 adults (mean [SD; range] age, 58.1 [1.1; 56-63] years; 1940 [44.7%] men and 2400 [55.3%] women). Of them, 630 (14.4%) were Black, 900 (20.7%) were Latinx, 2610 (61.0%) were White, and 210 (4.8%) were other race and ethnicity. The Table includes the descriptive statistics for demographic factors and medical morbidities by race and ethnicity for the participants included in the current analyses. Age and sex distributions were similar across race and ethnicity groups. A greater percentage of White participants completed college (790 [30.2%]) and graduate school (620, [23.8%]) than Black (140 [22.1%] and 110 [17.8%], respectively) and Latinx (190, [21.5%] and 120 [13.7%], respectively) participants. A greater percentage of Black (350 [55.6%]) and Latinx (340 [38.2%]) participants than White participants (810 [31.2%]) reported histories of hypertension as well as diabetes (150 [24.0%] of Black participants; 180 [20.5%] of Latinx participants; and 310 [12.0%] of White participants). Higher percentages of Black and Latinx participants had high BMI (380 [58.0%] of Black participants, 400 [44.5%] of Latinx participants, and 920 [35.5%] of White participants). A greater percentage of White participants reported a history of cancer (60 [9.3%] of Black participants, 100 [10.6%] of Latinx participants, and 440 [16.7%] of White participants) and had high cholesterol (70 [11.6%] of Black participants, 110 [12.6%] of Latinx participants, and 450 [17.3%] of White participants). Black participants (20 [3.7%]) were more likely to report a history of CKD than White (70 [2.5%]) and Latinx (20 [2.5%]) participants. Black participants were more likely to have 1 or 2 *APOE ε4* alleles (250 [38.7%]) than Latinx (180 [19.8%]) and White (700 [26.7%]) participants.

**Table.** Demographic Characteristic and Medical Comorbidity by Race and Ethnicity^a^

Characteristic	Participant Race and ethnicity, No. (%)					Total sample, weighted B value (95% CI)^c^
	White	Black	Latinx	Other^b^	Total sample	
No. of observations	2610 (60.1)	630 (14.4)	900 (20.7)	210 (4.8)	4340 (100)	NA
Demographic variables						
Age, mean (SD), y	58.0 (1.1)	58.4 (1.2)	58.3 (1.2)	58.1 (1.1)	58.1 (1.1)	58.2 (58.2-58.2)
Men	1180 (45.3)	260 (41.6)	400 (44.7)	100 (47.4)	1940 (44.7)	51.9 (51.0-52.9)
Women	1430 (54.7)	370 (58.4)	500 (55.3)	110 (52.6)	2400 (55.3)	48.1 (47.1-49.0)
Educational attainment						
High school or less	360 (13.6)	110 (17.6)	180 (20.1)	30 (13.9)	680 (15.6)	22.6 (21.4-23.7)
Some college	840 (32.3)	270 (42.6)	400 (44.7)	70 (31.1)	1580 (36.3)	43.6 (42.4-44.8)
College	790 (30.2)	140 (22.1)	190 (21.5)	60 (29.7)	1180 (27.2)	21.8 (20.8-22.8)
Graduate school	620 (23.8)	110 (17.8)	120 (13.7)	50 (25.4)	910 (20.9)	12.1 (11.2-12.9)
Medical morbidity						
CKD	70 (2.5)	20 (3.7)	20 (2.5)	10 (2.9)	120 (2.7)	2.6 (2.3-3.0)
Hypertension	810 (31.2)	350 (55.6)	340 (38.2)	60 (30.4)	1560 (36.1)	35.0 (33.6-36.3)
Diabetes	310 (12.0)	150 (24.0)	180 (20.5)	40 (17.9)	680 (15.8)	13.8 (12.8-14.8)
Cancer	440 (16.7)	60 (9.3)	100 (10.6)	20 (7.2)	600 (13.9)	14.2 (13.4-15.1)
Heart disease	100 (4.0)	30 (4.7)	40 (4.7)	10 (4.8)	180 (4.3)	4.3 (3.8-4.8)
High cholesterol	450 (17.3)	70 (11.6)	110 (12.6)	30 (14.0)	660 (15.4)	16.7 (15.1-18.2)
BMI in 2021-2022^d^						
Normal	730 (28.1)	90 (13.8)	180 (20.0)	70 (35.6)	1070 (24.7)	25.2 (23.9-26.5)
Elevated	950 (36.5)	180 (28.1)	320 (35.5)	70 (31.7)	1510 (34.8)	35.3 (34.0-36.5)
High	920 (35.5)	360 (58.0)	400 (44.5)	70 (32.7)	1750 (40.4)	39.6 (38.1-41.1)
*APOE ε4* status						
*APOE ε4* = 0	1910 (73.3)	380 (61.3)	720 (80.2)	160 (76.9)	3160 (73.2)	72.6 (70.8-74.5)
*APOE ε4* = 1	650 (24.9)	210 (33.1)	170 (18.5)	50 (21.6)	1060 (24.6)	24.4 (22.7-26.2)
*APOE ε4* = 2	50 (1.8)	40 (5.6)	10 (1.3)	<10 (1.4)	100 (2.2)	2.9 (2.1-3.8)

Abbreviations: BMI, body mass index; CKD, chronic kidney disease; NA, not applicable.

^a^ SOURCE: Author calculations are from the US Department of Education, National Center for Education Statistics, The High School and Beyond 1980: 2021 Follow-Up. Sample sizes are rounded and based on unweighted numbers.

^b^ Other included any other race or ethnicity.

^c^ Total sample (weighted) column provides statistics adjusted for population representativeness.

^d^ BMI calculated as [(weight in pounds)/(height in inches)^2^] × 703 and categorized as normal (<25.0), elevated (25.0-29.9), and high (≥30.0).

**Figure 2.**
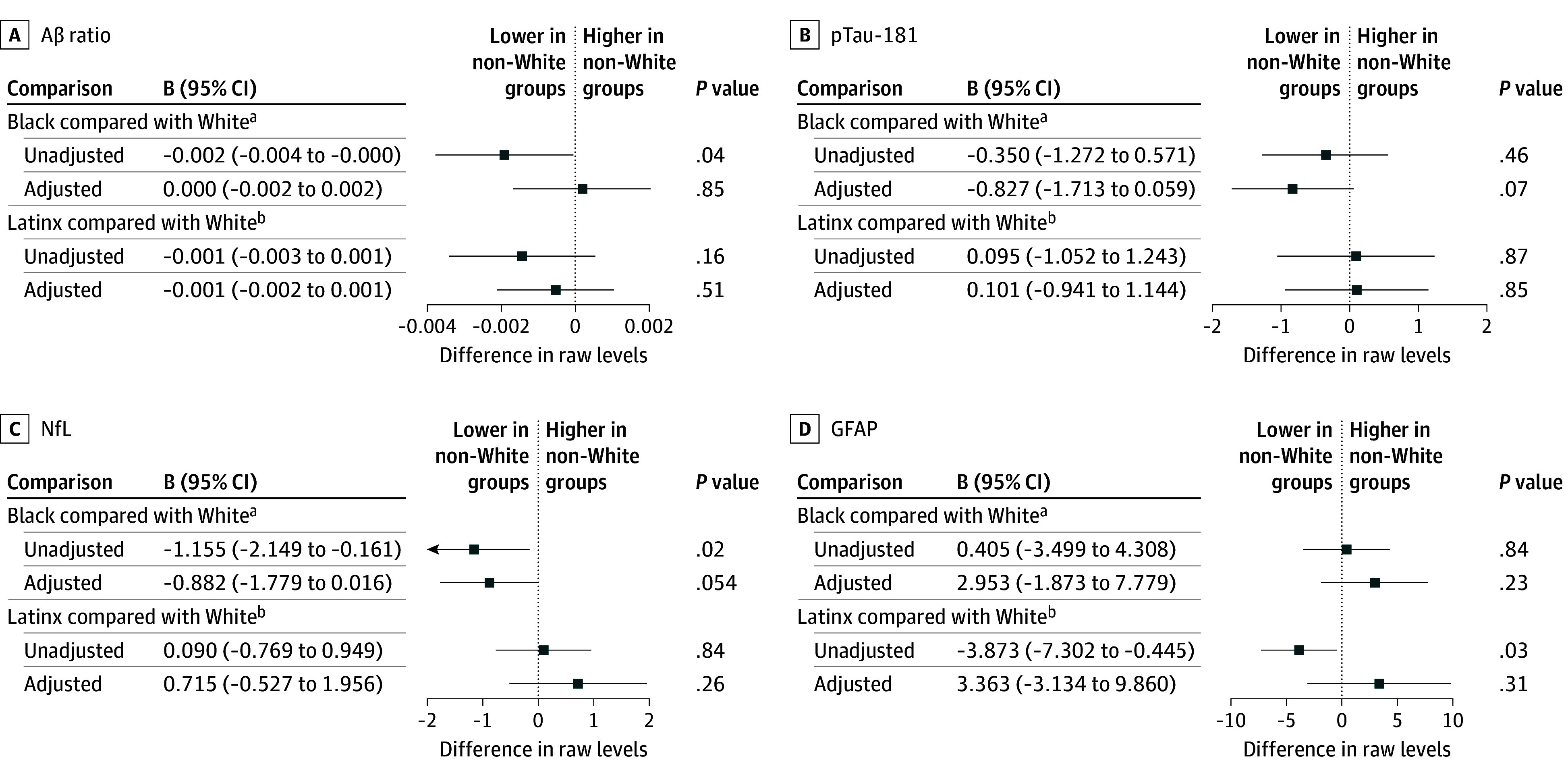
Comparison of Biomarker Concentrations Across Race and Ethnicity for Values That Are Unadjusted or Adjusted With Inverse Probability Weighting and Multiple Imputation B values show differences in raw biomarker levels (non-White groups minus White group); 95% CIs are adjusted for sampling weights. Aβ indicates amyloid-β; GFAP, glial fibrillary acidic protein; NfL, neurofilament light chain; pTau-181, phosphorylated tau-181. SOURCE: Author calculations are from the US Department of Education, National Center for Education Statistics, The High School and Beyond 1980: 2021 Follow-Up.

eFigure 4 in Supplement 1 displays distributions of raw and log transformed biomarker concentrations across race and ethnicity groups. Figure 2 displays differences between race and ethnicity groups in mean biomarker concentrations for raw means and for means that were estimated with inverse probability weighting and multiple imputation. eTable 2 in Supplement 1 displays biomarker concentrations by race and ethnicity. Without adjusting for the original sample design, sample attrition, and declination to provide a blood sample, Black participants had lower Aβ ratios and NfL concentrations than White participants (*d* = –0.002; 95% CI, –0.004 to –0.000; *P* = .04 for Aβ ratio and *d* = –1.16; 95% CI, –2.15 to –0.16; *P* = .02 for NfL). After adjusting estimates for population representation, these differences were attenuated and no longer statistically significant (*d* = 0.000; 95% CI, –0.002 to 0.002; *P* = .85 for Aβ ratio; *d* = −0.88; 95% CI, –1.78 to 0.02; *P* = .05 for NfL). Latinx participants had lower GFAP concentrations than White participants when considering unadjusted means (*d* = −3.87; 95% CI, –7.30 to –0.45; *P* = .03); similar to the observed Black compared with White participant differences, these differences were attenuated and no longer statistically significant when we adjusted estimates for population representation (*d* = 3.36; 95% CI, –3.13 to 9.86; *P* = .31). In supplemental analyses that included individuals with reported history of CKD, the general pattern of results remained the same, with the small and expected influence of CKD on the coefficients (eTable 3 in Supplement 1 for race and ethnicity comparisons of individuals without (Panel A) or with (Panel B) a history of CKD included).

**Figure 3.**
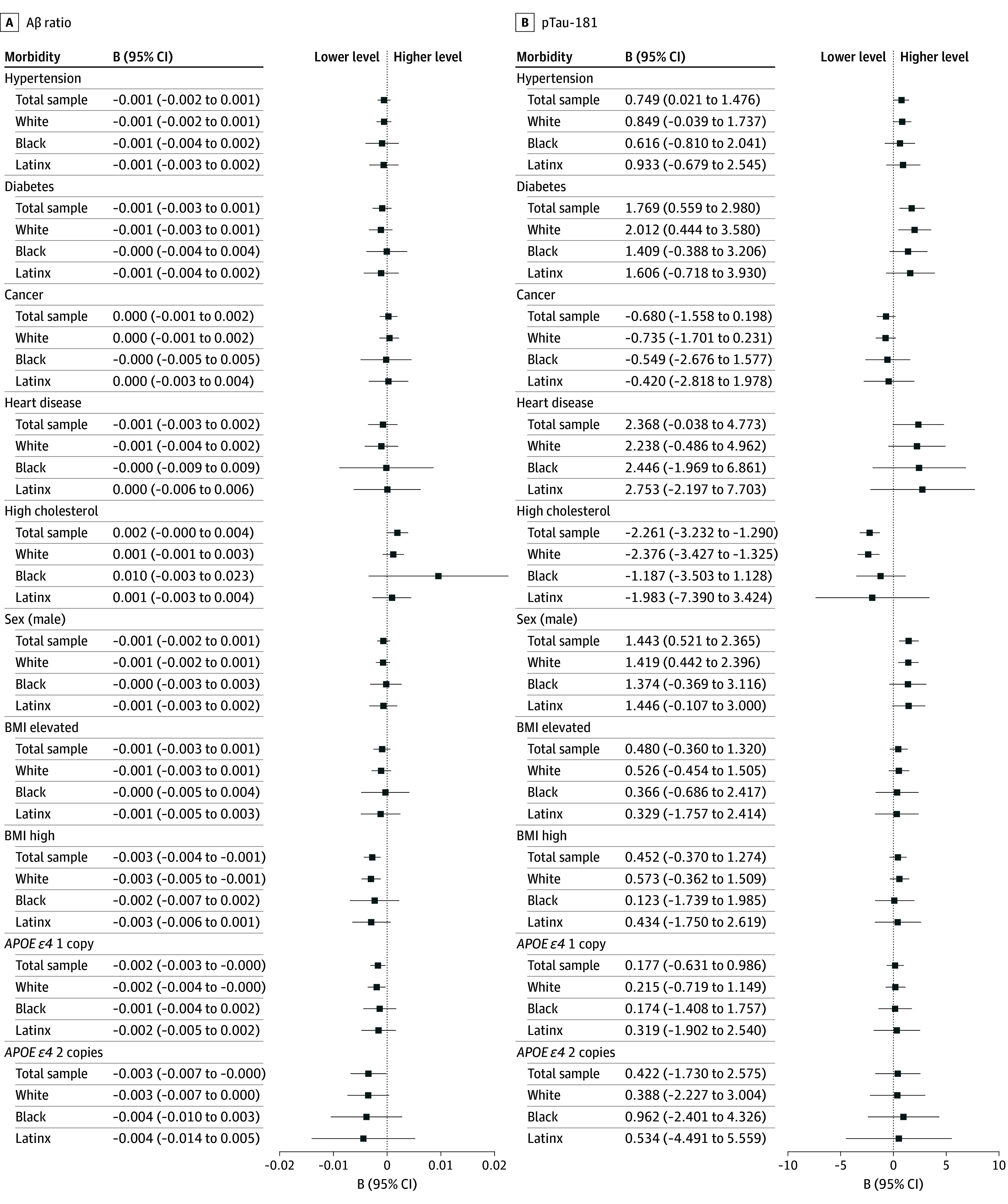
Differences in Biomarker Concentrations Between Individuals With or Without Medical Morbidities in the Total Sample and for Black, Latinx, and White Participants B values show differences in estimated population mean biomarker levels (with condition minus without condition); 95% CIs adjusted for population representativeness. Sex compared with female. BMI indicates body mass index, calculated as [(weight in pounds)/(height in inches)^2^] × 703 and categorized as normal (<25.0), elevated (25.0-29.9), and high (≥30.0); categories compared with normal BMI. *APOE ε4* categories compared with 0 copies. Aβ indicates amyloid-β; pTau-181, phosphorylated tau-181. SOURCE: Author calculations are from the US Department of Education, National Center for Education Statistics, The High School and Beyond 1980: 2021 Follow-Up.

**Figure 4.**
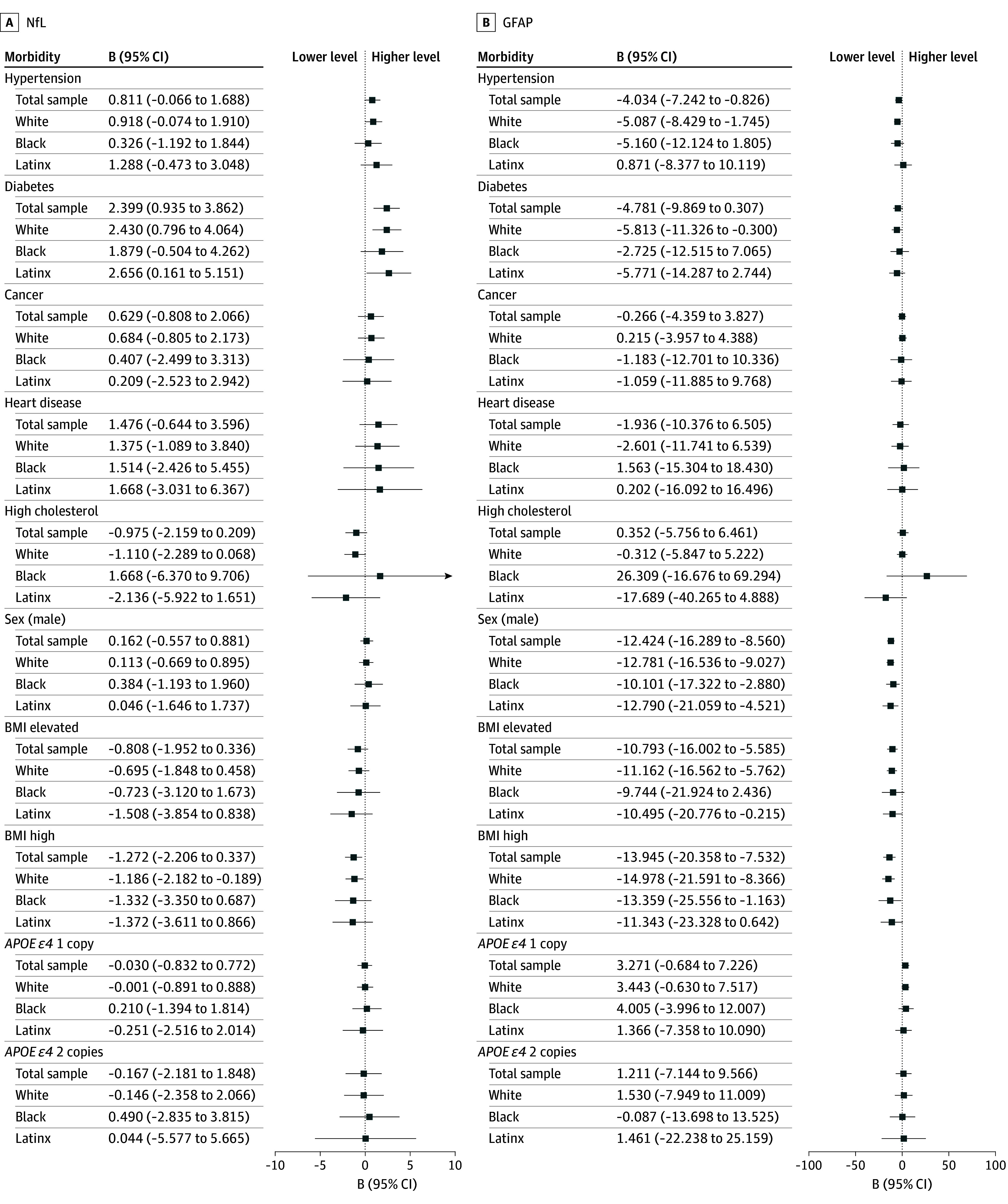
Differences in Biomarker Concentrations Between Individuals With or Without Medical Morbidities in the Total Sample and for Black, Latinx, and White Participants B values show differences in estimated population mean biomarker levels (with condition minus without condition); 95% CIs adjusted for population representativeness. Sex compared with female. BMI indicates body mass index, calculated as [(weight in pounds)/(height in inches)^2^] × 703 and categorized as normal (<25.0), elevated (25.0-29.9), and high (≥30.0); categories compared with normal BMI. *APOE ε4* categories compared with 0 copies. GFAP indicates glial fibrillary acidic protein; NfL, neurofilament light chain. SOURCE: Author calculations are from the US Department of Education, National Center for Education Statistics, The High School and Beyond 1980: 2021 Follow-Up.

Figure 3 and Figure 4 illustrate population-representative estimates for the association of sex, *APOE* status, and medical morbidities with biomarker concentrations in the total sample and separately for race and ethnicity groups. Statistical tests indicated differences across race and ethnicity groups were statistically indistinguishable in the associations of biomarkers with these characteristics (eTable 4 in Supplement 1). Based on raw *P* values, diabetes was associated with slightly higher concentrations of pTau-181 and hypertension was associated with lower concentrations of GFAP; however, these associations were not robust to controlling the false discovery rate (FDR) (*d* = 0.14; 95% CI, 0.04 to 0.23; *P* = .05 for diabetes with pTau-181; and *d* = −0.09; 95% CI, –0.16 to –0.02; *P* = .14 for hypertension with GFAP) (eTable 5 in Supplement 1). In contrast, diabetes was associated with higher concentrations of NfL (*d* = 0.19; 95% CI, 0.07-0.30; *P* = .04), and high cholesterol was associated with lower concentrations of pTau-181 (*d* = −0.18; 95% CI, −0.25 to −0.10; *P* = .01) with FDR adjustment. Women had higher GFAP values than men (*d* = 0.27; 95% CI, 0.19-0.35; *P* = .01) and lower pTau-181 values (*d* = −0.11; 95% CI, −0.04 to −0.19; *P* = .04) with FDR adjustment. Elevated BMI was associated with lower GFAP concentrations (*d* = −0.23; 95% CI, –0.35 to −0.12; *P* = .01), and high BMI was associated with lower Aβ ratios (*d* = −0.13; 95% CI, −0.21 to −0.06; *P* = .02) and lower GFAP values (*d* = −0.30; 95% CI −0.44 to −0.16; *P* = .01). Nominally, individuals with *APOE ε4* homozygosity had lower Aβ ratios than those with no *ε4* alleles, although this association did not persist with FDR control (*d* = −0.16; 95% CI, −0.32 to −0.01; *P* = .28).

## Discussion

In this nationally representative cohort study of middle-aged adults who were enrolled in high school in the US in 1980, we found that ADRD biomarker concentrations were similar across race and ethnicity groups when estimates were derived with models that relied on population representative data. We also found that common medical conditions, such as type 2 diabetes, high cholesterol, and elevated BMI, were associated with AD biomarker concentrations, but that these associations were similar across race and ethnicity groups among middle-aged adults and that the effect sizes were of small magnitude. Our findings have important implications for the characterization of ADRD pathophysiology and, potentially, diagnosis in diverse adults who represent the US population.

Without adjusting for population representativeness, Black participants had lower Aβ ratio values (indicating greater amyloid pathophysiology) and NfL concentrations (indicating lower levels of neurodegeneration) compared with White participants. Similarly, Latinx participants had lower concentrations of GFAP, a marker of astrocytosis or inflammation, compared with White participants. However, when we derived estimates from models adjusted to ensure population representation, these differences were attenuated and no longer statistically significant. A major strength of the HS&B:80 study is that the sampling scheme assembled a cohort representative of the population of high school students in 1980. We adjusted for inclusion in the initial panel sample with a high degree of confidence because probabilities of inclusion were known by design. Previous studies reported differences in blood-based biomarker levels among race and ethnicity groups,^9,10,11,12,13,14^ but these studies did not recruit based on population characteristics or did not ensure population representation statistically.

The approach we used to obtain population-representative estimates has important implications for understanding the mixed findings in the literature and suggest methodological considerations in this research area. When considering previously reported findings of race associated with differences in blood biomarker concentrations, the minimum detectable differences between race groups in our study at 80% power were less than the unadjusted differences in the group mean concentrations reported by Hajjar and colleagues,^12^ a study similar to ours in design and sample age. This observation suggests that even if there are minimal differences in the population means across race groups in middle age, they are substantially smaller than previously reported. Further, members of our team showed previously^32^ race and ethnicity differences in cognitive test performance in episodic memory and language. Results from the present study suggest that these differences may not be attributable, at least at the group level, to differences in AD pathophysiology as indexed by blood biomarker concentrations. We hypothesize that the observed race and ethnicity related differences in cognitive test performance in this age stratum reflect social determinants of health and their associations with non-AD related pathways.

There has been a concerted effort in recent years to increase the diversity of participants in observational and interventional research related to ADRD. However, the distinction between diversity in a research sample and its representation is critical, as associations with race and ethnicity in samples that are not population representative may not generalize.^33^ Increasing participation in research studies from minoritized groups does not ensure that those studies will be representative of those group; thus, racial and ethnic group comparisons could be misleading.

Our study highlights the possibility that medical comorbidities such as high BMI and diabetes may contribute to AD pathophysiology in middle age, particularly by having an impact on Aβ and neurodegeneration, reflected in Aβ ratio and NfL concentration, consistent with previous reports.^15^ Findings regarding the association of vascular factors with markers of AD pathophysiology have been mixed.^34^ In middle-aged adults, such as the age group included in this study, the impact of vascular factors with ADRD outcomes may be more prominent than in later life, when more severe AD neuropathology may obscure these associations. This possibility is consistent with emerging evidence that cerebrovascular disease is associated with ADRD plasma biomarker concentrations among individuals at genetic risk for AD, particularly when they are presymptomatic.^35^ Interestingly, individuals with high cholesterol had lower pTau-181 concentrations than those without high cholesterol, which may reflect the impact of cholesterol-lowering treatment. High BMI was associated with lower levels of GFAP, a finding that was unexpected but also consistent with 2 previous reports.^15,36^ Notably, however, the association of common medical comorbidities with biomarker concentrations did not differ significantly among race and ethnicity groups in this age group. While it is possible that differences in base rates of these medical comorbidities may ultimately contribute to race and ethnicity disparities in ADRD,^37^ they do not appear to have a differential impact on AD biomarker concentrations in middle age. We observed the well-documented^38,39,40^ higher frequency of the *APOE ε4* allele among Black individuals compared with Latinx or White individuals. Despite the differences in frequency, the associations between *APOE* genotype and plasma biomarker concentrations did not differ across race groups, which is also consistent with previous observations^41^ and suggests that the differences in *APOE ε4* frequency does not account for ADRD disparities in later life.

### Strengths and Limitations

Our study has several strengths. The large, prospectively recruited cohort that was population representative in 1980 enabled us to apply appropriate statistical techniques to ensure population representation of our findings. Unlike other large cohort studies, HS&B:80 is fixed within a specific age stratum, which avoids confounding associations with age or overadjustment for outcomes that are somewhat age dependent. The examination of individuals in late middle age allows for the study of the earliest manifestations of ADRD pathophysiology, when intervention may be most impactful and disparities may begin to emerge. This age group has not routinely been included in studies of aging and dementia.

Weaknesses of the study include the simultaneous measurement of medical morbidities and biomarkers, which limited our ability to draw conclusions about causal associations between medical morbidities and AD pathophysiology; ideally, biomarkers and medical variables should be collected at multiple time points to observe change over time. Further, the observed differences in outcome measures by medical factors were small and may not have been clinically meaningful, although we argue that any statistically reliable associations with appropriate adjustment observed may become amplified in older groups. While study of a middle-aged cohort is an overall strength, our findings may be more representative of earlier than preclinical AD, when divergence in AD pathophysiology may not be evident. In a similar vein, these biomarkers may be most sensitive at advanced stages of disease (not just age), so true predictive capability may be low in this age group. Finally, pTau-217 is now a more widely used biomarker than the pTau-181 marker we used in our study; however, previous research showed that blood concentrations of pTau-181 and pTau-217 are highly correlated with each other^42^ and likely capture tau-related pathophysiology similarly.

## Conclusions

In this population-representative cohort study of middle-aged adults (mean age approximately 58 years), we did not find evidence of differences in AD blood-based biomarker levels across race and ethnicity groups. Common medical conditions were reliably associated (albeit with small effect sizes) with biomarker concentrations similarly across race and ethnicity groups. These results highlight the importance of considering population representation and comorbid conditions in ADRD research to ensure accurate characterization of disease pathophysiology and improve precision of diagnostic and treatment strategies for populations that experience ADRD disparities. Our findings do not support the idea that different diagnostic thresholds should be applied to biomarker concentrations across race and ethnicity groups. Future studies should examine the association of plasma biomarker concentrations with cognitive function, cognitive impairment status, and future cognitive decline across race and ethnicity groups.

## Notes

Supplement 1. **eTable 1.** Variables used in multiple imputation **eTable 2.** ADRD biomarker concentrations by race and ethnicity (n = 4340) **eTable 3.** Primary analyses showing unweighted and weighted race and ethnicity comparisons among individuals without a reported history of CKD (Panel A) and with individuals with a reported CKD included (Panel B) **eTable 4.** Joint test for differences across race and ethnicity groups in associations between biomarkers and medical morbidities **eTable 5.** Relationships between biomarker concentrations and medical morbidities in the total sample (n = 4210) **eFigure 1.** Imputation diagnostic plots: distribution of observed and imputed biomarker values **eFigure 2.** Imputation diagnostic plots: trace plots for the means and standard deviations of biomarkers across 10 imputations **eFigure 3.** Associations of ADRD biomarker concentration with creatine and self-reported kidney disease **eFigure 4.** Distribution of biomarker concentrations for raw and log transformed values across race and ethnicity groups (n = 4340)

Supplement 2.
Data Sharing Statement
