# Supplementary material for: Alzheimer Disease Blood Biomarker Concentrations Across Race and Ethnicity Groups in Middle-Aged Adults
Source: JAMA Netw Open. 2025 Nov 21;8(11):e2545046. doi: 10.1001/jamanetworkopen.2025.45046 (PMC12639488; doi:10.1001/jamanetworkopen.2025.45046)
Supplement: Supplement 1. — eTable 1. Variables used in multiple imputation eTable 2. ADRD biomarker concentrations by race and ethnicity (n = 4340) eTable 3. Primary analyses showing unweighted and weighted race and ethnicity comparisons among individuals without a reported history of CKD (Panel A) and with individuals with a reported CKD included (Panel B) eTable 4. Joint test for differences across race and ethnicity groups in associations between biomarkers and medical morbidities eTable 5. Relationships between biomarker concentrations and medical morbidities in the total sample (n = 4210) eFigure 1. Imputation diagnostic plots: distribution of observed and imputed biomarker values eFigure 2. Imputation diagnostic plots: trace plots for the means and standard deviations of biomarkers across 10 imputations eFigure 3. Associations of ADRD biomarker concentration with creatine and self-reported kidney disease eFigure 4. Distribution of biomarker concentrations for raw and log transformed values across race and ethnicity groups (n = 4340) [file jamanetwopen-e2545046-s001.pdf]

Supplementary Online Content

Brickman AM, Muller C, Warren JR, et al. Alzheimer disease blood biomarker concentrations across race and ethnicity groups in middle-aged adults. *JAMA Netw Open*. 2025;8(11):e2545046. doi:10.1001/jamanetworkopen.2025.45046

eTable 1. Variables used in multiple imputation

eTable 2. ADRD biomarker concentrations by race and ethnicity (n=4,340)

eTable 3. Primary analyses showing unweighted and weighted race and ethnicity comparisons among individuals without a reported history of CKD (Panel A) and with individuals with a reported CKD included (Panel B)

eTable 4. Joint test for differences across race and ethnicity groups in associations between biomarkers and medical morbidities

eTable 5. Relationships between biomarker concentrations and medical morbidities in the total sample (n=4,210)

eFigure 1. Imputation diagnostic plots: distribution of observed and imputed biomarker values

eFigure 2. Imputation diagnostic plots: trace plots for the means and standard deviations of biomarkers across 10 imputations

eFigure 3. Associations of ADRD biomarker concentration with creatine and self-reported kidney disease

eFigure 4. Distribution of biomarker concentrations for raw and log transformed values across race and ethnicity groups (n=4,340)

This supplementary material has been provided by the authors to give readers additional information about their work.

© 2025 Brickman AM et al. *JAMA Network Open*.

eTable 1. Variables used in multiple imputation

- Variables from 2021:
  - General cognition score
  - Educational attainment
  - Employment status
  - Marital status
  - Self-reported health
  - BMI
  - ApoE carrier status
  - Smoking
  - Kessler psychological distress score
  - Science knowledge score
  - Self-reported cancer diagnosis
  - Self-reported hypertension diagnosis
  - Self-reported stroke diagnosis
  - Self-reported cardiovascular disease diagnosis
  - Self-reported mental health diagnosis
  - Self-reported periodontal disease diagnosis
  - Self-reported kidney disease diagnosis
  - Self-reported diabetes diagnosis
  - High cholesterol (from lipid panel)
  - ADRD biomarkers
  - Creatinine
  - Urea Nitrogen
- Variables from 2014/15:
  - Employment status
  - Marital status
  - Self-reported health
  - Kessler psychological distress score
- Variables from high school:
  - Parental education
  - Parental income
  - Parents owned home
  - Socio-economic status index
  - Physical disability
  - High school program

© 2025 Brickman AM et al. *JAMA Network Open*.

    - Highest level math course taken
    - High school grade point average (GPA)
    - Academic achievement test scores
    - School value added score
- Additional variables:
    - Study cohort (sophomore or senior)
    - High school region
    - High school urbanicity
    - High school type (public, private, Catholic)
    - Sex
    - Race/ethnicity
    - Marital status in 1986 and 1992
    - Indicators of post-secondary educational experience between 1982-1992

© 2025 Brickman AM et al. *JAMA Network Open*.

eTable 2. ADRD biomarker concentrations by race and ethnicity (n=4,340)

	Race/ethnicity				
	White	Black	Hispanic	Other	Total sample
	2,610<br>60.1%	630<br>14.4%	900<br>20.7%	210<br>4.8%	4,340 100.0%
Amyloid beta 42:40 ratio	0.059 (0.018)	0.059 (0.020)	0.058 (0.017)	0.056 (0.025)	0.059 (0.018)
pTau-181 (pg/mL)	18.4 (12.7)	17.1 (9.6)	19.1 (14.8)	19.2 (9.6)	18.4 (12.6)
Neurofilament light chain (pg/mL)	14.2 (14.9)	12.5 (12.0)	15.7 (16.5)	15.8 (12.2)	14.2 (14.7)
Glial fibrillary acidic protein (pg/mL)	69.8 (46.8)	71.1 (49.2)	73.7 (74.4)	67.5 (35.8)	70.2 (49.8)

Notes: Data were adjusted for population representativeness. Units: Aβ ratio (ratio); Ptau-181, NfL, and GFAP (pg/mL). Standard deviations are reported in the parentheses. All sample sizes are rounded to the nearest 10 per NCES restricted use data license requirements.

SOURCE: Author calculations from U.S. Department of Education, National Center for Education Statistics, The High School & Beyond 1980: 2021 Follow-Up. Sample sizes are rounded and based on unweighted numbers.

© 2025 Brickman AM et al. *JAMA Network Open*.

eTable 3. Primary analyses showing unweighted and weighted race and ethnicity comparisons among individuals without a reported history of CKD (Panel A) and with individuals with a reported CKD included (Panel B). Panel C displays the differences in coefficients (n=4,340).

	Amyloid beta 42:40 ratio	Ptau-181 (pg/mL)	Neurofilament light chain (pg/mL)	Glial fibrillary acidic protein (pg/mL)	Amyloid beta 42:40 ratio	Ptau-181 (pg/mL)	Neurofilament light chain (pg/mL)	Glial fibrillary acidic protein (pg/mL)
	Unweighted				Weighted			
Panel A: Without CKD								
Black	-0.002*	-0.350	-1.155*	0.405	0.000	-0.827	-0.882	2.953
	(0.001)	(0.469)	(0.506)	(1.989)	(0.001)	(0.449)	(0.455)	(2.437)
Latinx	-0.001	0.095	0.090	-3.873*	-0.001	0.101	0.715	3.363
	(0.001)	(0.585)	(0.438)	(1.747)	(0.001)	(0.527)	(0.627)	(3.279)
Others	-0.000	0.626	1.562	-2.049	-0.001	0.254	1.190	1.072
	(0.002)	(0.845)	(1.065)	(2.550)	(0.002)	(1.001)	(1.012)	(3.781)
N	4,120	4,200	4,120	4,120	4,120	4,200	4,120	4,120
Panel B: With CKD								
Black	-0.002	-0.370	-0.926	0.622	0.000	-0.801	-0.777	3.036
	(0.001)	(0.479)	(0.541)	(1.979)	(0.001)	(0.463)	(0.476)	(2.440)
Latinx	-0.001	0.494	0.655	-4.080*	-0.001	0.119	0.787	3.395
	(0.001)	(0.669)	(0.581)	(1.722)	(0.001)	(0.557)	(0.656)	(3.271)

© 2025 Brickman AM et al. *JAMA Network Open*.

Others	-0.000	0.829	1.920	-1.934	-0.001	0.204	1.196	1.107
	(0.002)	(0.896)	(1.150)	(2.528)	(0.002)	(0.994)	(1.034)	(3.825)
N	4,240	4,330	4,240	4,240	4,240	4,330	4,240	4,240
Panel C: Difference in coefficients								
Black	0	0.02	-0.229	-0.217	0	-0.026	-0.105	-0.083
Latinx	0	-0.399	-0.565	0.207	0	-0.018	-0.072	-0.032
Others	0	-0.203	-0.358	-0.115	0	0.05	-0.006	-0.035

Standard errors in parentheses. * p<0.05, ** p<0.01, *** p<0.001.

SOURCE: Author calculations from U.S. Department of Education, National Center for Education Statistics, The High School & Beyond 1980: 2021 Follow-Up. Sample sizes are rounded and based on unweighted numbers.

© 2025 Brickman AM et al. *JAMA Network Open*.

eTable 4. Joint test for differences across race and ethnicity groups in associations between biomarkers and medical morbidities

	Aβ ratio			pTau-181			NfL			GFAP		
	d.f.	F-stat	p-value	d.f.	F-stat	p-value	d.f.	F-stat	p-value	d.f.	F-stat	p-value
Hypertension	2	0.024	0.976	2	0.054	0.948	2	0.428	0.652	2	0.657	0.519
Diabetes	2	0.147	0.863	2	0.148	0.862	2	0.142	0.868	2	0.199	0.820
Cancer	2	0.028	0.972	2	0.040	0.961	2	0.062	0.940	2	0.050	0.951
Heart Disease	2	0.062	0.940	2	0.020	0.980	2	0.007	0.993	2	0.154	0.857
High Cholesterol	2	0.815	0.443	2	0.426	0.653	2	0.372	0.690	2	1.883	0.153
Male	2	0.062	0.940	2	0.003	0.997	2	0.071	0.932	2	0.363	0.696
BMI Normal	2	0.022	0.979	2	0.065	0.937	2	0.107	0.899	2	0.104	0.901
BMI Elevated	2	0.116	0.890	2	0.022	0.978	2	0.351	0.704	2	0.382	0.683
BMI High	2	0.016	0.984	2	0.123	0.885	2	0.082	0.921	2	0.381	0.683
APOE e4=0	2	0.035	0.965	2	0.005	0.995	2	0.080	0.923	2	0.102	0.903
APOE e4=1	2	0.090	0.914	2	0.012	0.988	2	0.052	0.949	2	0.125	0.883
APOE e4=2	2	0.025	0.976	2	0.042	0.959	2	0.048	0.953	2	0.040	0.961

Notes: Data were adjusted for population representativeness. Analyses exclude individuals with chronic kidney disease. Units: Aβ ratio (ratio); Ptau-181, NfL, and GFAP (pg/mL). F-statistic test whether the relationship between the biomarker and morbidity differs for Black and Hispanic individuals related to White individuals. "d.f." refers to the degrees of freedom associated with the test, and the p-value for the corresponding F-test. SOURCE: Author calculations from U.S. Department of Education, National Center for Education Statistics, The High School & Beyond 1980: 2021 Follow-Up. Sample sizes are rounded and based on unweighted numbers.

© 2025 Brickman AM et al. *JAMA Network Open*.

eTable 5. Relationships between biomarker concentrations and medical morbidities in the total sample (n=4,210)

	Aβ ratio					pTau-181					NfL					GFAP				
	Coef.	Std Coef.	s.e.	Raw p	FDR p	Coef.	Std Coef.	s.e.	Raw p	FDR p	Coef.	Std Coef.	s.e.	Raw p	FDR p	Coef.	Std Coef.	s.e.	Raw p	FDR p
Hypertension	-0.001	-0.027	0.028	0.336	0.73	0.749	0.059	0.029	0.044	0.28	0.811	0.063	0.034	0.070	0.33	-4.034	-0.088	0.035	0.014	0.14
Diabetes	-0.001	-0.044	0.042	0.291	0.67	1.769	0.138	0.048	0.004	0.05	2.399	0.185	0.057	0.002	0.04	-4.781	-0.104	0.056	0.065	0.33
Cancer	0.000	0.014	0.039	0.724	0.99	-0.680	-0.053	0.035	0.128	0.48	0.629	0.049	0.056	0.388	0.81	-0.266	-0.006	0.045	0.898	0.99
Heart Disease	-0.001	-0.036	0.065	0.584	0.99	2.368	0.185	0.095	0.054	0.32	1.476	0.114	0.083	0.171	0.56	-1.936	-0.042	0.093	0.651	0.99
High Cholesterol	0.002	0.089	0.048	0.065	0.33	-2.261	-0.177	0.039	0.000	0.01	-0.975	-0.075	0.047	0.106	0.45	0.352	0.008	0.068	0.910	0.99
Male	-0.001	-0.033	0.029	0.255	0.67	1.443	0.113	0.036	0.003	0.04	0.162	0.013	0.028	0.656	0.99	-12.424	-0.270	0.042	0.000	0.01
BMI Elevated	-0.001	-0.046	0.037	0.218	0.65	0.480	0.038	0.033	0.261	0.67	-0.808	-0.062	0.045	0.164	0.54	-10.793	-0.234	0.057	0.000	0.01
BMI High	-0.003	-0.132	0.038	0.001	0.02	0.452	0.035	0.033	0.279	0.67	-1.272	-0.098	0.036	0.008	0.09	-13.945	-0.303	0.070	0.000	0.01
APOE e4=1	-0.002	-0.081	0.035	0.020	0.18	0.177	0.014	0.032	0.666	0.99	-0.030	-0.002	0.031	0.941	0.99	3.271	0.071	0.043	0.104	0.45
APOE e4=2	-0.003	-0.163	0.080	0.044	0.28	0.422	0.033	0.085	0.699	0.99	-0.167	-0.013	0.079	0.870	0.99	1.211	0.026	0.092	0.775	1.00

Notes: Data were adjusted for population representativeness. Analyses exclude individuals with chronic kidney disease. BMI categories compared to normal BMI (<25). APOE4 categories compared to 0 copies. Sex compared to women. Units: Aβ ratio (ratio); Ptau-181, NfL, and GFAP (pg/mL). False discovery rate (FDR) p-values adjusted using the Benjamini-Hochberg procedure for all statistical tests in this study. SOURCE: Author calculations from U.S. Department of Education, National Center for Education Statistics, The High School & Beyond 1980: 2021 Follow-Up. Sample sizes are rounded and based on unweighted numbers.

© 2025 Brickman AM et al. *JAMA Network Open*.

eFigure 1. Imputation diagnostic plots: distribution of observed and imputed biomarker values. Kernel density plots for the observed (solid line) and imputed (dashed line) biomarker values. Distributions are truncated at the 99th percentile for visualization clarity (n=4,340).

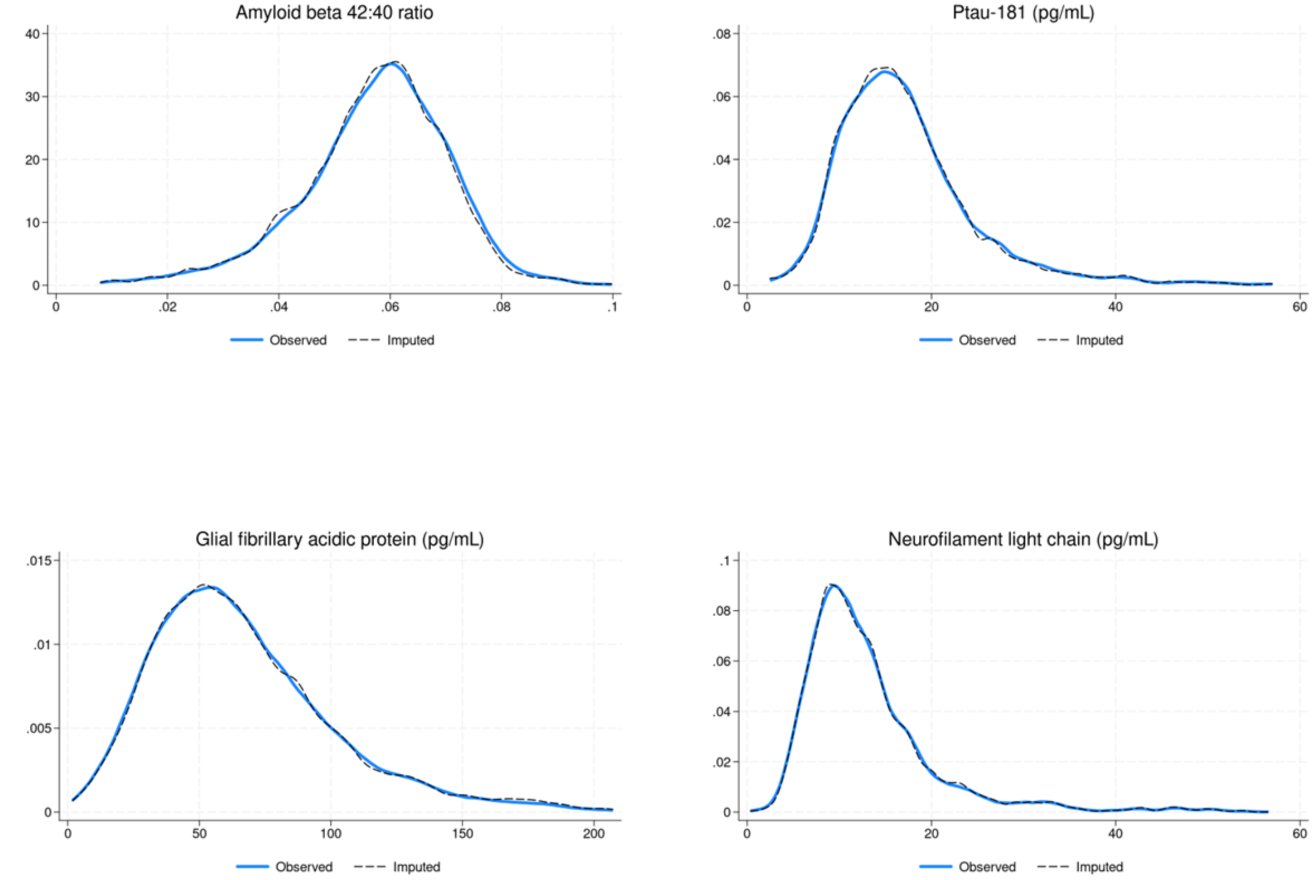

SOURCE: Author calculations from U.S. Department of Education, National Center for Education Statistics, The High School & Beyond 1980: 2021 Follow-Up. Sample sizes are rounded and based on unweighted numbers.

© 2025 Brickman AM et al. *JAMA Network Open*.

eFigure 2. Imputation diagnostic plots: trace plots for the means and standard deviations of biomarkers across 10 imputations. The plots display the estimated means and standard deviations for each biomarker over 10 iterations. Each line represents a different imputation (n=4,340).

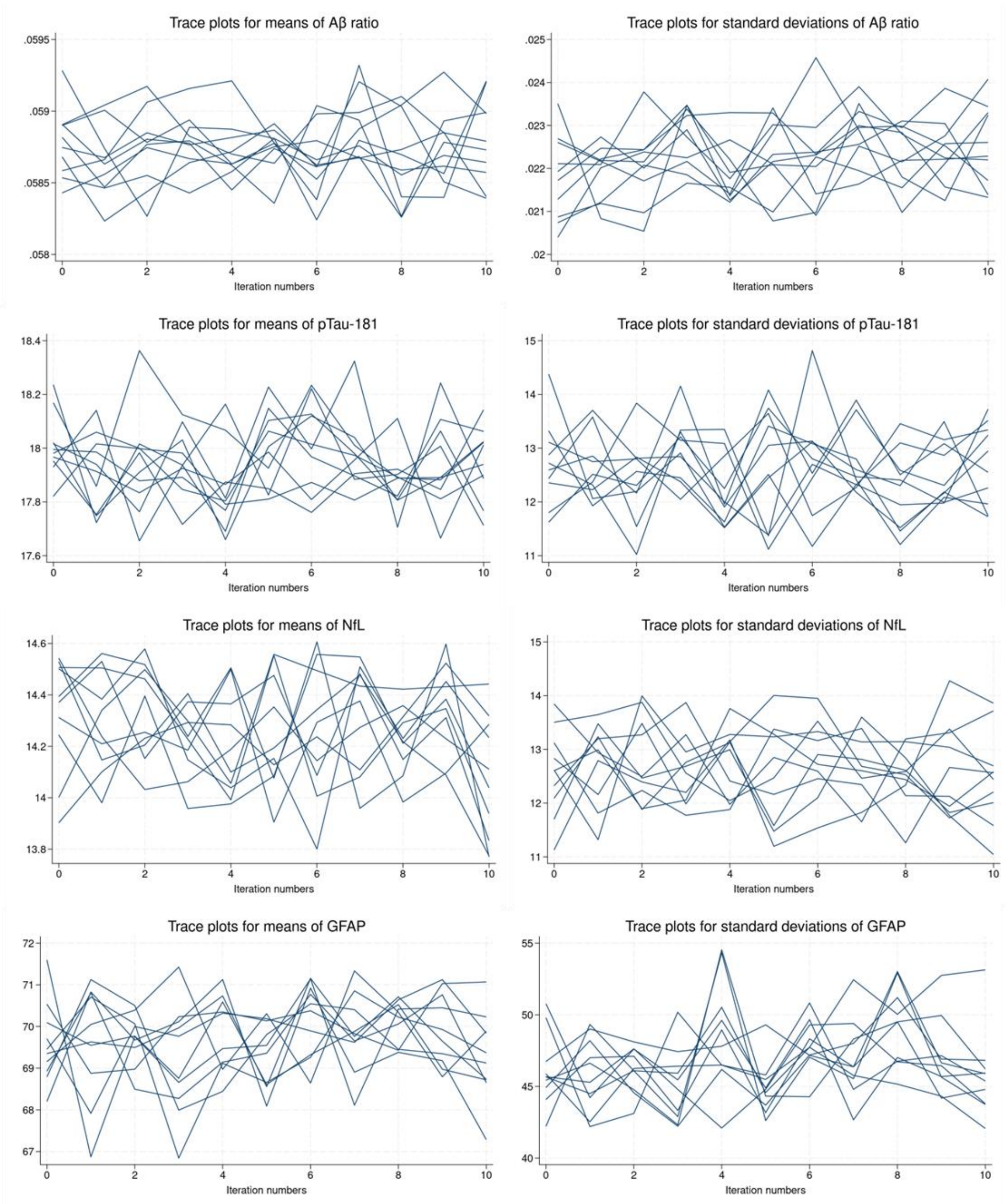

SOURCE: Author calculations from U.S. Department of Education, National Center for Education Statistics, The High School & Beyond 1980: 2021 Follow-Up. Sample sizes are rounded and based on unweighted numbers.

© 2025 Brickman AM et al. *JAMA Network Open*.

eFigure 3. Associations of ADRD biomarker concentration with creatine and self-reported kidney disease. Individuals with reported history of CKD had higher levels of all biomarker outcomes; similarly, creatinine levels were positively correlated with all biomarker concentrations. Those with a history of CKD (n=130) were removed from subsequent analyses (n=4,340).

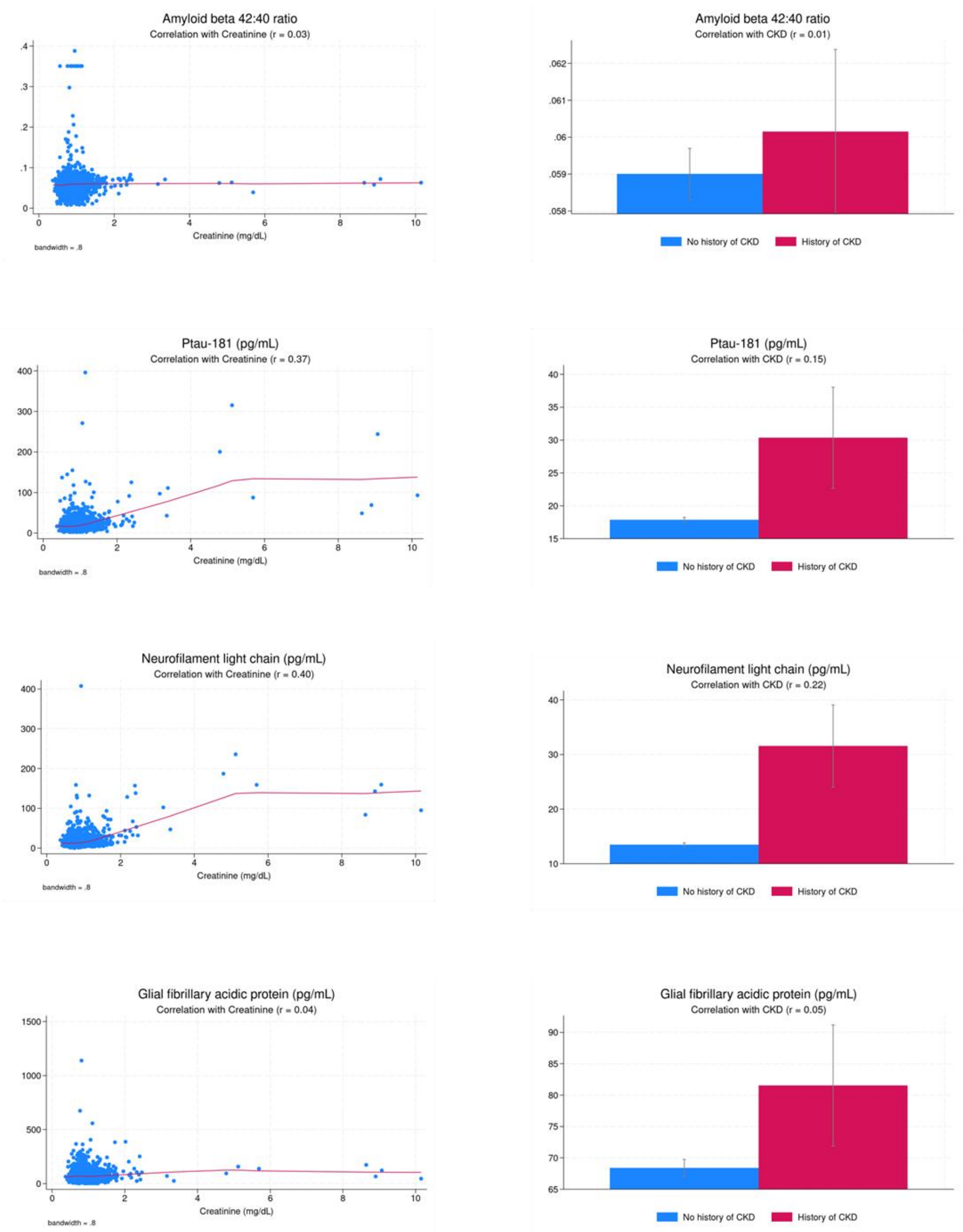

SOURCE: Author calculations from U.S. Department of Education, National Center for Education Statistics, The High School & Beyond 1980: 2021 Follow-Up. Sample sizes are rounded and based on unweighted numbers.

© 2025 Brickman AM et al. *JAMA Network Open*.

eFigure 4. Distribution of biomarker concentrations for raw and log transformed values across race and ethnicity groups (n=4,340)

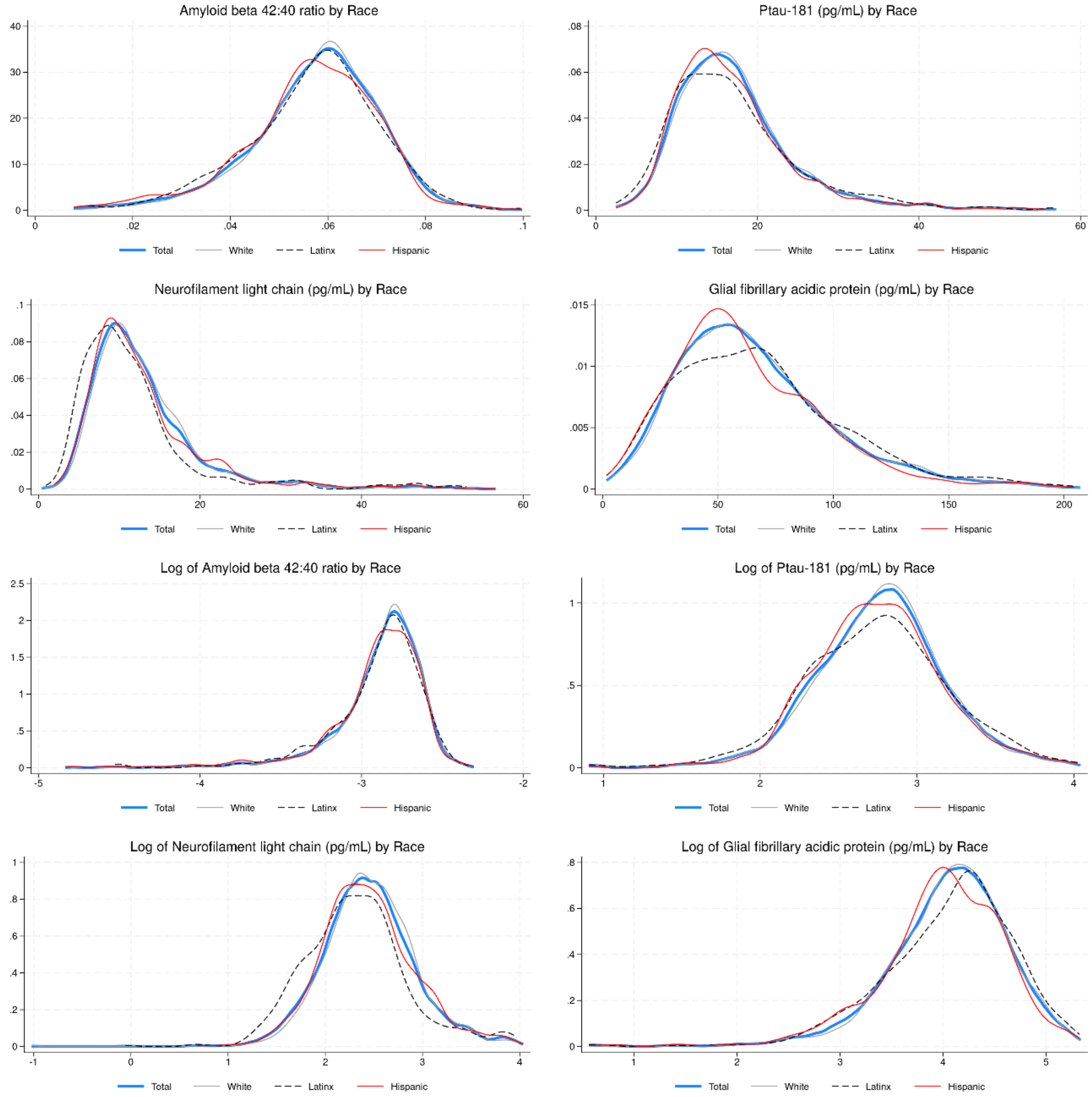

SOURCE: Author calculations from U.S. Department of Education, National Center for Education Statistics, The High School & Beyond 1980: 2021 Follow-Up. Sample sizes are rounded and based on unweighted numbers.

© 2025 Brickman AM et al. *JAMA Network Open*.
