# Supplementary material for: Alzheimer Disease Blood Biomarker Concentrations Across Race and Ethnicity Groups in Middle-Aged Adults
Source: JAMA Netw Open. 2025 Nov 21;8(11):e2545046. doi: 10.1001/jamanetworkopen.2025.45046 (PMC12639488; doi:10.1001/jamanetworkopen.2025.45046)
Supplement: Supplement 2. — Data Sharing Statement [file jamanetwopen-e2545046-s002.pdf]

Data Sharing Statement

Brickman. Alzheimer Disease Blood Biomarker Concentrations Across Race and Ethnicity Groups in Middle-Aged Adults. *JAMA Netw Open*. Published November 21, 2025. doi:10.1001/jamanetworkopen.2025.45046

Data
Data available: Yes
Data types: Deidentified participant data
How to access data: The data used in the current study are under the jurisdiction of the Institute of Education Sciences, U.S. Department of Education (https://ies.ed.gov/about/restricted-use-data). Qualified users can apply for access to the data.
When available: With publication

Supporting Documents
Document types: None

Additional Information
Who can access the data: Qualified investigators can apply for a license to access the data.
Types of analyses: Upon review
Mechanisms of data availability: No support will be provided. Users will require a license to access data through the Institute of Education Sciences, U.S. Department of Education (https://ies.ed.gov/about/restricted-use-data), although terms are currently being negotiated. Please refer to https://edshareproject.org/ for updated information about data sharing.
